# The increasing use of the WHO Safe Childbirth Checklist: lessons learned at the Yaoundé Gynaeco-Obstetric and Paediatric Hospital, Cameroon

**DOI:** 10.1186/s12884-021-03966-4

**Published:** 2021-07-08

**Authors:** Julius Sama Dohbit, Namanou Ines Emma Woks, Carlin Héméry Koudjine, Willy Tafen, Pascal Foumane, Assumpta Lucienne Bella, Rosemary Nkemdilim Ogu, Fru Fobuzshi Angwafo

**Affiliations:** 1 Faculty of Medicine and Biomedical Sciences, University of Yaoundé I, Yaoundé, Cameroon; 2 Yaoundé Gynaeco-Obstetric and Paediatric Hospital (YGOPH), Yaoundé, Cameroon; 3 Faculty of Clinical Sciences, University of Port Harcourt, Port Harcourt, Nigeria

**Keywords:** Safe childbirth, Checklist, World Health Organization, Cameroon

## Abstract

**Background:**

Safe childbirth remains a daunting challenge, particularly in low-middle income countries, where most pregnancy-related deaths occur. Cameroon’s maternal mortality rate, estimated at 529 per 100,000 live births in 2017, is significantly high. The WHO Safe Childbirth Checklist (SCC) was designed to improve the quality of care provided to pregnant women during childbirth. The SCC was implemented at the Yaoundé Gynaeco-Obstetric and Paediatric Hospital to improve the quality of care during childbirth.

**Methods:**

This study was a retrospective study to determine the adoption rate of the SCC and its association with maternal (eclampsia, perineal tears, and postpartum haemorrhage) and neonatal (stillbirth, neonatal asphyxia and neonatal death) complications. Data were collected 6 months after the introduction of the SCC. Multivariate binary logistic regression was used to analyse the association between the use of the SCC and maternofoetal complications.

**Results:**

Out of 1611 deliveries conducted, 1001 records were found, giving a retrieval rate of 62%. Twenty-five records were excluded. During the study period, the checklists were used in 828 of 976 clinical notes, with an adoption rate of 84.8% and a utilization rate of 93.9% at 6 months. Severe preeclampsia/eclampsia was associated with the non-use of the SCC (2.1 vs 5.4%, *p* = 0.041). Stillbirth, neonatal asphyxia, and neonatal death rates were not significantly different between the checklist and non-checklist groups. However, for all neonatal outcomes, the proportion of complications was lower when the checklist was used.

**Conclusion:**

The use of the SCC was associated with significantly reduced pregnancy complications, especially for reducing the rates of severe pre-eclampsia/eclampsia. The use of the SCC increased to 93.9% of all deliveries within 6 months. We advocate for the use of the WHO Safe Childbirth Checklist in maternity units.

**Supplementary Information:**

The online version contains supplementary material available at 10.1186/s12884-021-03966-4.

## Background

Childbirth is a moment of overwhelming expectation and anxiety for the family as a whole and the couple in particular. Safe childbirth remains a daunting challenge, particularly in low-resource settings, where most pregnancy-related deaths occur. Achieving the desired reduction in the global maternal mortality ratio to less than 70 per 100,000 live births is one of the Sustainable Development Goals’ (SDG) healthcare targets [1].

Maternal mortality is a global health problem. In 2017, the World Health Organization (WHO) reported 295 000 maternal deaths due to pregnancy and childbirth complications [2]. Most of these women died of preventable causes in low-middle income countries (LMICs). Furthermore, for each case of maternal death, many more women experience life-threatening complications or severe maternal morbidity (SMM) [3]. Cameroon’s maternal mortality ratio (MMR), estimated at 529 per 100,000 live births in 2017, is still unacceptably high [4]. More than 50% of these deaths are due to haemorrhage, hypertensive disorders, and sepsis [5, 6].

The main factors responsible for SMM and preventable maternal deaths are limited access to care, poor quality services and poverty [7]. A multicountry study conducted in LMICs reported that closing the quality gap would produce substantial benefits in maternal and neonatal health at current levels of access and utilization of healthcare services [8]. Therefore, reducing maternal mortality and morbidity in Cameroon hinges on identifying and improving services that are critical to the health of women and girls, such as antenatal care, emergency obstetric and newborn care (EmONC), skilled birth attendance and quality healthcare before, during and after childbirth [9–13].

Poor quality of care (QoC) can occur because of the know-do gap, whereby healthcare providers’ actions diverge from the knowledge of evidence-based recommendations [14, 15]. According to the WHO, inadequacies in QoC provided in hospitals are responsible for unsatisfactory maternal and neonatal health indicators despite increased geographical coverage [16]. Consequently, maternity services need to ensure standard QoC to achieve expected health outcomes in women and their newborns [17]. Identifying and instituting cost-effective interventions that improve the quality and safety of childbirth is beneficial. Facility-based checklist interventions have been successfully used to help practitioners translate recommendations into high-quality patient care [18].

This WHO Safe Childbirth Checklist (SCC) is a facility-based reminder tool comprised of 28 essential birth practices (see Table 1), which target the major causes of maternal deaths, stillbirths and neonatal deaths [19, 20]. The SCC was designed to help health workers adhere to evidence-based practices associated with improved maternal and neonatal outcomes at critical moments of delivery. It consists of four pause points (or moments of care): on admission, just before pushing or performing a caesarean, within 1 h after birth, and shortly before discharge. A study conducted in India in 2016 showed significantly improved adherence to practices 6 months posttraining in the intervention arm [21]. Furthermore, the BetterBirth trial in India reported an increase in providers’ adherence to essential birth practices after 2 months of peer coaching in primary-level facilities [22]. However, there are limited studies on the use of SCC and the association between the use of SCC and pregnancy-related complications.

**Table 1:** List of 28 essential birth practices from the World Health Organization Safe Childbirth Checklist [19]

**List of Essential Birth Practices**
***On admission***
**1. Assess mother’s need for referral**
**2. Start partograph**
**3. Assess mother for criteria to start antibiotics**
**4. Assess mother for signs of pre-eclampsia/eclampsia or the need to administer magnesium sulphate and antihypertensive treatment**
**5. Ensure availability of supplies to clean hands and wear gloves for each vaginal exam confirmed**
**6. Encourage birth companion to be present at birth**
**7. Confirm that mother or companion will call for help during labour if needed**
***Just before pushing (or before caesarean)***
**8. Assess mother for criteria to start antibiotics**
**9. Assess mother for signs of pre-eclampsia/eclampsia or the need to administer magnesium sulphate and antihypertensive treatment**
**10. Confirm essential supplies for mother at bedside**
**11. Confirm essential supplies for baby bedside**
**12. Identify assistant and confirm readiness to help at birth if needed**
***Soon after birth (within 1 h)***
**13. Assess mother for abnormal bleeding**
**14. Assess mother for criteria to start antibiotics**
**15. Assess mother for signs of pre-eclampsia/eclampsia or the need to administer magnesium sulphate and antihypertensive treatment**
**16. Assess baby’s need for referral**
**17. Assess baby for criteria to start antibiotics**
**18. Assess baby’s needs for special care/monitoring**
**19. Initiate skin-to-skin contact and breastfeeding (if mother and baby are well)**
**20. Confirm that mother/companion will call for help if danger signs present**
***Before discharge***
**21. Confirm stay at facility for 24 h after delivery**
**22. Assess mother for criteria to start antibiotics**
**23. Assess mother’s blood pressure**
**24. Assess mother for abnormal bleeding**
**25. Assess baby for criteria to start antibiotics**
**26. Establish good breastfeeding practices before discharge**
**27. Discuss and offer family planning options to mother**
**28. Arrange follow-up and confirm mother/companion will seek help if danger signs are present after discharge**

Maternal and neonatal mortality rates are key indicators of the health system’s strength [23]. Implementing strategies with the potential to increase adherence to essential birth practices is necessary to prevent pregnancy-related complications and deaths in health facilities. The Yaoundé Gynaeco-Obstetric and Paediatric Hospital (YGOPH) thus introduced the SCC at her Obstetrics and Gynaecology Service. This study assessed SCC use and its association with obstetric and neonatal complications during the 6 months following its introduction and staff training at the YGOPH.

## Methods

### Aim of the study

#### Primary objective

To determine the adoption rate of SCC and its association with maternal and neonatal complications 6 months after its introduction at YGOPH, Cameroon.

#### Secondary objectives

To evaluate the completion rate of the SCC during the first 6 months of implementation at the YGOPH, Cameroon.

To assess the relationship between the SCC and the maternal and neonatal complications.

### Study design

A retrospective study at the Gynaecology and Obstetrics service of the YGOPH. Data were collected 6 months after the introduction of the SCC. Six months was chosen because previous research conducted on SCC in India [21] showed that adherence to essential birth practices could be evaluated within this period.

### Study setting

Cameroon has a pyramidal health system. YGOPH, a tertiary referral hospital specializing in maternal and child care, is at the summit of the pyramid. YGOPH has one of the busiest maternity units in the country, with over 3,000 deliveries annually and a 32% caesarean birth rate.

The Gynaecology and Obstetrics Service has three units: inpatient maternity, inpatient gynaecology and outpatient gynaecology. The service has 11 gynaecologists/obstetricians, one general practitioner and 56 nursing staff. Vaginal births are conducted at the maternity unit, made up of 13 admission beds. A total of 20 nursing staff work in the maternity unit. They comprise 16 skilled birth attendants (SBAs), one nurse specializing in reproductive health, one state registered nurse (SRN), one nursing assistant (NA) and one certified nurse (CN). Two of these (SRN and SBA) are unit heads at maternity.

### The characteristics of the participants

#### Inclusion criteria

All women who gave birth at the YGOPH from January to June 2018 with delivery records or clinical notes at the archives of Gynaecology and Obstetrics Service were assessed for eligibility. The clinical note or delivery record is the primary tool used to document care, communicate plans and provide guidance for follow-up treatment and care of each patient.

#### Exclusion criteria

- § Women with incomplete clinical notes (without the mode of delivery mentioned)
- § Women admitted to the maternity unit for observation or medical reasons (malaria during pregnancy, urinary infection during pregnancy, threatened preterm labour) without an ensuing delivery
- § Women who gave birth (delivered) before 22 weeks of gestation
- § Women who delivered in other facilities and were referred to the YGOPH in the postpartum period

#### Minimum sample size

All women who met the eligibility criteria were included in this study. A sample size calculation was performed using the formula below:

$$N = {\raise0.7ex\hbox{${\left[ {\left( {Z_{{ \propto /2}} } \right)^{2} P\left( {1 - P} \right)} \right]}$} \!\mathord{\left/ {\vphantom {{\left[ {\left( {Z_{{ \propto /2}} } \right)^{2} P\left( {1 - P} \right)} \right]} {d^{2} }}}\right.\kern-\nulldelimiterspace} \!\lower0.7ex\hbox{${d^{2} }$}}$$

P: adoption rate of SCC in a tertiary hospital in Sri Lanka [24]. *P* = 45.8%

d: degree of precision: d = 0.05.

$Z_{{ \propto /2}}$ = the standard normal variate (at a 5% type I error (*P* < 0.05), it is 1.96).

N = [(1.96)^2^ 0.458(1–0.458)]/(0.05)^2^ = 381 cases.

Adjusted minimum sample size: n' = (n)/(1 – q), where n' is the corrected minimum sample size, n is the initial minimum sample size, and q is the probable proportion of poorly filled questionnaires (10%).

The adjusted minimum sample size is **424.**

### Procedure

#### Implementation of the SCC (see Supplementary Figure 1)

The management of the Gynaecology and Obstetrics Service introduced the SCC programme in December 2017. The WHO SCC was used [19]. An illustrative presentation of how to use the SCC during childbirth was provided by an obstetrician at the monthly service meeting. Thereafter, pilot testing of the SCC commenced at the maternity unit. The nursing staff of the maternity unit used the SCC during the pilot phase from October—December 2017 under the daily supervision of the two maternity unit heads who informed the service heads weekly.

The official launching of the SCC was performed at a specially organized seminar in the first week of January 2018. During this 1-day workshop, the SCC was slightly modified, and a refresher course was given on how to use it. The modified SCC had the criterion for the administration of antibiotics after premature rupture of membranes changed from more than 18 h to more than 6 h. After this seminar, the maternity unit heads attached the SCC to the current and new delivery records of individual patients. Supervision continued daily by the unit heads and weekly by the hospital nursing directors.

#### Data collection tools

Data were collected from the women’s clinical notes and from the nurses providing care to the women. This paper presents the findings from the women’s clinical notes. The following information was gathered from the women’s clinical notes (with the complications defined as shown below):

- § Identification: code, age, place of residence
- § History: obstetric, surgical, medical
- § Completion rate of the checklist for each parturient and newborn
- § Clinical notes with checklists used and without checklists used

#### Definition of independent variables

- § **Use of the SCC:** Refers to the total number of SCCs partially and fully filled during care for each patient: on admission, just before pushing at childbirth or performing a caesarean delivery, 1 h after childbirth or caesarean delivery, and just before discharge.
- § **Completion rate of the SCC**: The number of fully filled SCCs used divided by the total number of partial and completely filled SCCs used.

#### Definition of specific maternal factors

##### Maternofoetal complications

This refers to pregnant women with severe pre-eclampsia & eclampsia, acute foetal distress (AFD), perineal tears, or post-partum haemorrhage (PPH).

- § **Mild pre-eclampsia:** The presence of a systolic blood pressure (SBP) greater than or equal to 140 mm Hg or a diastolic blood pressure (DBP) greater than or equal to 90 mm Hg or higher, occurring after 20 weeks’ gestation, in addition to a urine dipstick protein of 1 + **(**approximately 30 mg/dL), 2 + (100 mg/d) or more.
- § **Severe pre-eclampsia:** An SBP greater than or equal to 160 mm Hg or a DBP greater than or equal to 110 mm Hg or higher, occurring after 20 weeks’ gestation, in addition to a urine dipstick protein of 1 + **(**approximately 30 mg/dL), 2 + (100 mg/d) or more.
- § **Eclampsia:** The onset of grand mal seizures and/or unexplained coma during pregnancy or postpartum, during or after the 20th week of gestation in a woman with signs or symptoms of preeclampsia.
- § **A perineal tear:** A tear or injury to the skin and/or muscles between the vaginal introitus and the anal opening.
- § **Primary postpartum haemorrhage (PPH):** Blood loss of more than 500 ml from the genital tract: uterus, cervix, vagina and perineum within 24 h of delivery.
- § **Secondary PPH:** Any significant vaginal or uterine bleeding occurring between 24 h of delivery and 6 weeks **postpartum.**

#### Neonatal factors

- § **Stillbirth**: Foetal death, which occurred between 22 weeks gestation and the time of delivery.
- § **Neonatal asphyxia:** Persistence of an Apgar score of 1–6 at the 1^st^ and 5^th^ minutes following delivery.
- § **Neonatal death:** The death of a newborn to mothers included in the study within the first 7 days of life.

#### Definition of covariates

- § **Age:** Number of years lived by the woman.
- § **Parity:** The number of previous pregnancies experienced by a woman that have resulted in a live birth or a stillbirth.
- § **Primiparous**: A woman who has given birth to one live or dead foetus of ≥ 22 weeks gestation.
- § **Multiparous:** A woman who has given birth to more than one live or dead foetus of ≥ 22 weeks gestation.
- § **Gestational hypertension:** Hypertension (SBP greater than or equal to 140 mm Hg or a DBP greater than or equal to 90 mm Hg or higher) that occurs de novo, usually during the latter half of pregnancy in the absence of proteinuria and other signs and symptoms of preeclampsia.
- § **Chronic hypertension:** Patients who, before pregnancy, had an SBP greater than or equal to 140 mm Hg or a DBP greater than or equal to 90 mm Hg.

### Statistical analysis

Statistical analysis was performed using the SPSS version 23·0 software. Descriptive statistics were used to summarize the nominal data. The chi-square test was used to compare categorical variables. Multivariate binary logistic regression analysis was performed to eliminate confounders in the association between the use of the SCC and maternal and neonatal complications. The independent variable was the use of the checklist, while dependent variables were: maternofoetal complications, maternal complications, eclampsia/severe preeclampsia, perineal tears, post-partum haemorrhage, neonatal complications, neonatal asphyxia, stillbirth and neonatal deaths. The results were considered statistically significant at *P* < 0.05.

## Results

There were a total of 1611 births during the study period. Among their records, 1001 complete records were available, a 62% retrieval rate (See Supplementary Figure 1). Twenty-five records were excluded: seven abortions, nine pregnancy-related pathologies discharged before childbirth and nine cases referred from other facilities after childbirth. The mean age of our study population was 28 ± 6 years. The most represented age group was 25–29 years (see Table 2). Most of our study population was multiparous.

**Table 2:** Sociodemographic and clinical characteristics of the study population

	**Frequency (%)**
**Age**	
15–19 years	73 (7.5)
20–24 years	189 (19.4)
25–29 years	311 (31.9)
30–34 years	214 (21.9)
35–39 years	153 (15.7)
≥ 40 years	35 (3.6)
Missing data	1 (0.1)
**Total**	976 (100.0)
**Parity**	
1–2	520 (53.3)
3–4	217 (22.2)
≥ 5	81 (8.3)
Missing data	158 (16.2)
**Previous pathology in study population**	**Frequency (n)**
Chronic hypertension	8 (11.1)
Gestational hypertension	34 (47.2)
Gestational diabetes	2 (2.8)
PPH	3 (4.1)
Others	25 (34.7)
**Mode of birth**	**Frequency (n)**
Vaginal birth	807 (82.7)
Caesarean birth	169 (17.3)
**Total**	**976 (100.0)**

*PPH* Post-partum haemorrhage

Of the 976 records, a total of 828 included completed checklists, an adoption rate of 84.8% (See Table 3 for details). Furthermore, there was an increase in checklist adoption rate over time, with a peak of 93.9% in May and June. We documented the highest proportion of clinical notes with unused checklists in January and February (See Table 3). Three peripartum pathologies were common in the medical history of the parturient: gestational and chronic hypertension, gestational diabetes and postpartum haemorrhage. The most frequent pathology was gestational hypertension. Caesarean sections were the most frequent surgical procedures previously performed on the women.

**Table 3:** Proportion of checklist use and monthly completion rate of the checklist

Month	Groups		Proportion of checklist use	Completion rate of the checklist	Total
	**Checklist use**	**Non-checklist**			
**January**	110	53	67·5	2.7	163
**February**	162	48	77·1	6.2	210
**March**	122	13	90·4	1.6	135
**April**	113	13	89·7	13.3	126
**May**	130	8	94·2	8.5	138
**June**	191	13	93·6	20.9	204
Total	**828**	**148**	**84·8**	**/**	**976**
**Completion rate(%) of the checklist at different pause points**					
	**On admission**	**Just before delivery**	**Within 1 h of delivery**	**Before discharge**	
January	**27.3**	**2.7**	**4.5**	**12.7**	
February	**37.7**	**1.2**	**8.0**	**24.1**	
March	**36.1**	**1.6**	**3.3**	**49.2**	
April	**54.0**	**3.5**	**7.1**	**49.6**	
May	**48.5**	**3.1**	**4.6**	**62.3**	
June	**61.8**	**52.9**	**20.4**	**62.8**	

The completion rate of the SCC just before delivery and within 1 h of birth was consistently below 10% during the first 5 months. However, by the sixth month of the study, more than 60% of the checklists were properly filled on admission and before discharge.

**Fig. 1:**
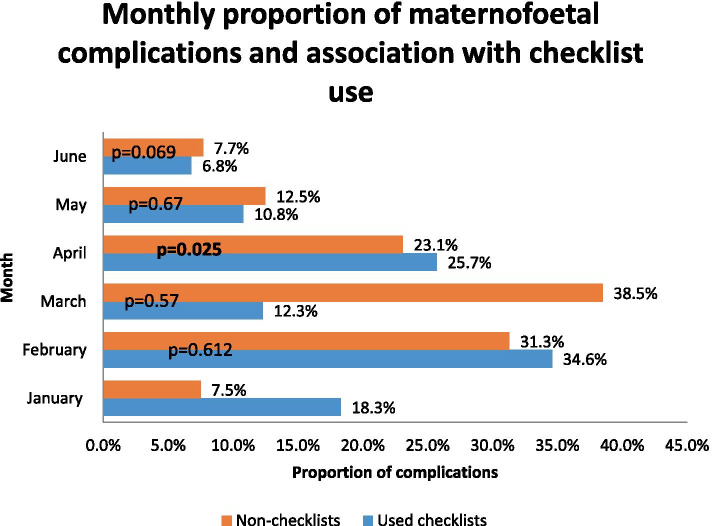
Monthly proportion of maternofoetal complications and association with checklist use

Of 976 delivery records, the YGOPH registered 176 patients with maternofoetal complications during the study as shown in Table 4. The difference in the proportion of maternofoetal complications between checklist and non-checklist cases was not statistically significant (147 vs 29; *p* = 0.566) (see Table 4). A monthly analysis revealed that the percentage of adverse maternofoetal outcomes rose during the first 4 months from 18.3 to 25.7% and then dropped to 6.8% during the last month among cases with used checklists (See Fig. 1). A significant reduction in the onset of severe pre-eclampsia/eclampsia was associated with the use of checklists (2.1 vs 5.4%, *p* = 0.041). Conversely, there was no significant difference between the checklist and non-checklist categories for patients with neonatal asphyxia, perineal tears or PPH.

**Table 4:** Association between the use of the checklist and maternofoetal complications

**Variables**	**Checklist use (N1 = 828)**	**Non-checklist group (N2 = 148)**	**Total**	***P*-value**
**Maternofoetal complications**				
Yes	147 (17.8)	29 (19.7)	176 (18.1)	0.566
No	681 (82.2)	118 (80.3)	799 (81.9)	
**Maternal complications**				
Yes	83 (10.0)	14 (9.5)	97 (9.9)	0.832
No	745 (90.0)	134 (90.5)	879 (90.1)	
**Eclampsia/Severe pre-eclampsia**				
Yes	17 (2·1%)	8 (5·4%)	25 (2·6%)	**0.041**
No	811 (97·9%)	140 (94·6%)	951 (97·4%)	
**Perineal tears**				
Yes	59 (7.1%)	6 (4.1%)	65 (6.7%)	0.167
No	769 (92.9%)	142 (95.9%)	911 (93.3%)	
**Post-partum Haemorrhage**				
Yes	8 (1)	0	8 (0.8)	0.615
No	820 (99)	148 (100)	968 (99.2)	
**Neonatal complications**				
Yes	70 (8.5)	15 (10.2)	85 (8.7)	0.491
No	757 (91.3)	132 (89.8)	889 (91.3)	
**Neonatal asphyxia**				
Yes	37 (4.5)	8 (5.4)	45 (4.6)	0.619
No	790 (95.5)	140 (94.6)	930 (95.4)	
**Stillbirth**				
Yes	33 (4)	7 (4.7)	40 (4.1)	0.676
No	794 (96.0)	141 (95.3)	935 (95.9)	
**Neonatal deaths**				
Yes	2 (0.2)	1 (0.7)	3 (0.3)	0.39
No	825 (99.8)	147 (99.3)	972 (99.7)

Table 4 highlights the frequency of neonatal complications encountered during the study and its association with SCC use. Among the 85 clients with neonatal complications, 40 (4.1% of births) had stillbirths, 45 (4.6%) developed neonatal asphyxia, and three (0.3%) died within the first 48 h of life. Nevertheless, neonatal asphyxia and neonatal death rates were not significantly different between the checklist and non-checklist groups.

## Discussion

This study assessed the use of SCC at YGOPH and its association with maternal and neonatal complications during the 6-month period following SCC implementation. The mean age of our study population was 28 ± 6 years. Of the 976 delivery records, 828 used the checklists. Severe preeclampsia/eclampsia was associated with the non-use of the SCC (2.1 vs 5.4%, *p* = 0.041).

In this study, the adoption rate of the SCC over a 6-month period was 84.8% (828/976 files). This value is twice as high as the adoption rate (45.8%) reported in a tertiary care setting in Sri Lanka during a 2-month prospective observational study conducted in 2013 [24]. At the YGOPH, there were approximately 270 deliveries done per month; meanwhile, at De Soysa Women’s Hospital (DSWH), in Sri Lanka, the number of births conducted overtime was significantly higher (400 births/month). Therefore, the greater workload and short duration of the study at the DSWH could have contributed to this difference in adoption rates. Additionally, a deeper commitment to quality improvement by the staff of YGOPH may explain the higher checklist adoption rate, since by the sixth month (June), almost all deliveries (93.9%) were managed with a filled childbirth checklist. Another study conducted in a district hospital in Rwanda also found high essential birth practice (EBP) compliance at 56% over a 2-month period following staff training of SCC implementation [25].

Our evaluation found that the SCC-based intervention at the YGOPH was associated with a significantly lower proportion (2.1 vs 5.4%, *p* = 0.041) of severe preeclampsia/eclampsia cases. Regarding the mechanism by which this occurred, the SCC prompts the birth attendant to check the blood pressure on admission (see Table 1) and, if indicated, commence prophylaxis with magnesium sulphate. This early blood pressure check ensures early identification and management, thus preventing deterioration to severe pre-eclampsia/eclampsia. This hypothesis has been validated by a quasi-experimental study [26] performed in Rajasthan, India, which observed a positive behavioural change in health care providers associated with the implementation of the SCC. The greatest difference (64%) was found in the early identification, management and timely referral of cases of preeclampsia [26]. Although the Better-Birth study, a cluster-randomized, controlled trial in Uttar Pradesh, India, found no significant impact of the SCC intervention on maternal morbidity or mortality [27], the unavailability of medications and consumables may have been responsible. Although both studies are based on the SCC, they differ in context. The Better-Birth study facilities were a combination of primary health care facilities and community health centres, whereas YGOPH is a tertiary-level facility. We posit that in the context of adequate human and material resources, the use of the SCC will be associated with a significant reduction in maternal morbidity and mortality.

After multivariate analysis shown in Table 5 and Supplementary Table 1, the significant variation in the proportion of severe pre-eclampsia and eclampsia cases in both SCC groups was maintained when the results were adjusted for differences in age, parity and a history of hypertension.

**Table 5:** Multivariate analysis of maternofoetal complications associated with the non-use of the checklist

**Dependent variables**	**Independent variable (Checklist use)**		**Adjusted OR**	**CI**	***P*-value**
	**Yes (N1 = 828)**	**No (N2 = 148)**			
**Maternofoetal complications**					
Yes	147 (17.8%)	29 (19.7%)	0.91	0.58–1.42	0.664
No	681 (82.2%)	118 (80.3%)			
**Maternal complications**					
Yes	83 (10.0%)	14 (19.5%)	1.14	0.62–2.08	0.676
No	745 (90.0%)	134 (90.5%)			
**Eclampsia/Severe pre-eclampsia**					
Yes	17 (2.1%)	8 (5.4%)	0.41	0.17–0.99	**0.049**
No	811 (97.9%)	140 (94.6%)			
**Perineal tears**					
Yes	59 (7.1%)	6 (4.1%)	1.895	0.80–4.50	0.147
No	769 (92.9%)	142 (95.9%)			
**Neonatal complications**					
Yes	70 (8.5%)	15 (10.2%)	0.81	0.45–1.46	0.487
No	757 (91.5%)	132 (89.8%)			
**Neonatal asphyxia**					
Yes	37 (4.5%)	8 (5.4%)	0.81	0.45–1.46	0.487
No	790 (95.5%)	140 (94.6%)			
**Stillbirth**					
Yes	33 (4.0%)	7 (4.7%)	0.809	0.37–1.78	0.598
No	794 (96.0%)	141 (95.3%)			
**Neonatal deaths**					
Yes	2 (0.2%)	1 (0.7%)	0.375	0.03–4.18	0.425
No	825 (99.8%)	147 (99.3%)			

The multivariate analysis results were adjusted for age, parity, chronic and gestational hypertension

Figure 1 shows a progressive decline in the proportion of maternal complications with the use of the SCC over time. The checklists were attached to the clinical notes. The percentage of maternofoetal complications dropped to 6.8% during the last month among cases with a completed checklist. As described in previous studies [21, 22], the use of the SCC along with regular coaching or supervision improves adherence to essential birth practices, thereby resulting in fewer complications when providers have adequate skills combined with the availability of essential supplies.

Nevertheless, neonatal asphyxia and neonatal death rates were not significantly different between the checklist and non-checklist groups, as shown in Table 4. However, for all neonatal outcomes, the proportion of complications was lower when the checklist was used. Thus, we agree with other published works [16, 28, 29] that non-adherence to essential birth practices affects the quality of care and consequently neonatal outcomes.

## Study limitations

This study is a review of outcomes during implementation research in one facility to provide better quality of care during childbirth. We had no influence on the quality of data entered into the delivery records. However, measures taken to minimize this limitation were compared with the data in the delivery registers and service reports. This study was only carried out in one facility.

Out of 1611 deliveries conducted during the study period, 1001 delivery records were found, a retrieval rate of 62%. We currently have a paper-based archiving system, and one of the limitations associated with this is the loss of files in the patient record circuit. However, the hospital is in the process of computerizing their medical records to eradicate the problem of missing records.

## Conclusion and recommendations

The use (adoption rate) of the SCC increased to 93.9% of all deliveries within 6 months of implementation. Our study showed that the utilization of the SCC was associated with a significant reduction in the onset of severe preeclampsia/eclampsia. We advocate for the use of the SCC in maternity units. Based on the positive results obtained, we intend to continue using this reminder tool and encourage other health facilities to use it as well.

## Perspectives

The hospital is transitioning to electronic medical records to enable better archival of its medical data.

## Supplementary Information

**Additional file 1: Supplementary figure 1.** SCC introduction flowchart at the YGOPH.

**Additional file 2: Supplementary table 1a and 1b.** Details of multivariate analysis (a) *and *(b).

## Data Availability

The datasets generated and/or analysed during the current study are available from the corresponding authors on request.

## Abbreviations

EBP: Essential birth practices
HGOPY/YGOPH: Yaoundé Gynaeco-Obstetric and Paediatric Hospital
SCC: Safe Childbirth Checklist
WHO: World Health Organization
