# Supplementary material for: The increasing use of the WHO Safe Childbirth Checklist: lessons learned at the Yaoundé Gynaeco-Obstetric and Paediatric Hospital, Cameroon
Source: BMC Pregnancy Childbirth. 2021 Jul 8;21:497. doi: 10.1186/s12884-021-03966-4 (PMC8268572; doi:10.1186/s12884-021-03966-4)
Supplement: Supplementary file 1 — Additional file 1: Supplementary figure 1. SCC introduction flowchart at the YGOPH. [file 12884_2021_3966_MOESM1_ESM.docx]

Supervision

- Labour & post-partum wards survey at least once daily
- Watch how the SCC is used
- Collect reports at daily nursing handover shifts on challenges related to the use of the SCC

Oct -Dec 2017: Introduction of the SCC

SCC program introduced at the maternity of the YGOPH.

- Illustrative presentation by Obstetrician

Pilot testing use of the SCC at the maternity

Early January 2018: Official launching of SCC

Seminar to officially launch the SCC: one-day session

Data collection from delivery records of January to June 2018

(n=1611 deliveries)

(n=1001 records found)

Early July  2018: Data collection

Excluded (n=25)

- 7 abortions
- 9 discharged cases prior to childbirth
- 9 post-delivery admissions

Data analyses and reporting

Continuous use of the SCC at the maternity

August 2018 : Data analyses

Supplementary figure 1: SCC’s Introduction flowchart at the YGOPH
