# Supplementary material for: The increasing use of the WHO Safe Childbirth Checklist: lessons learned at the Yaoundé Gynaeco-Obstetric and Paediatric Hospital, Cameroon
Source: BMC Pregnancy Childbirth. 2021 Jul 8;21:497. doi: 10.1186/s12884-021-03966-4 (PMC8268572; doi:10.1186/s12884-021-03966-4)
Supplement: Supplementary file 2 — Additional file 2: Supplementary table 1a and 1b. Details of multivariate analysis (a) and (b). [file 12884_2021_3966_MOESM2_ESM.docx]

**Supplementary table 1a: Details of multivariate analysis (a)**

| Dependent variables and covariates | checklist use(SCC) | | | | Adjusted OR | CI (95%) | P-value |
| --- | --- | --- | --- | --- | --- | --- | --- |
|  | **Total** | | **Yes** | **No** |  |  |  |
| MATERNOFOETAL COMPLICATIONS | | | | | | | |
| Yes | 176 | 147(17.8) | | 29(19.7) | 0.91 | 0.58-1.42 | 0.664 |
| No | 799 | 681(82.2) | | 118(80.3) |  |  |  |
| Age |  |  | |  |  |  |  |
| ≤19 years | 73 | 61(7.4) | | 12(8.1) | 1.06 | 0.58-2.05 | 0.858 |
| >19 years | 902 | 766(92.6) | | 136(91.9) |  |  |  |
| Parity |  |  | |  |  |  |  |
| Primiparous | 463 | 386(46.6) | | 77(52.0) | 1.35 | 0.90-2.04 | 0.152 |
| Multiparous | 513 | 442(53.4) | | 71(48.9) |  |  |  |
| Past history of gestational hypertension |  |  | |  |  |  |  |
| Yes | 34 | 26(3.1) | | 8(5.4) | 1.77 | 0.75-4.19 | 0.194 |
| No | 942 | 802(96.9) | | 140(94.6) |  |  |  |
| Past history of chronic hypertension |  |  | |  |  |  |  |
| Yes | 8 | 6(0.7) | | 2(1.4) | 1.42 | 0.26-7.87 | 0.689 |
| No | 965 | 820(99.3) | | 145(98.6) |  |  |  |
| MATERNAL COMPLICATIONS | | | | | | | |
| Yes | 97 | 83(10.0) | | 14(19.5) | 1.14 | 0.62-2.08 | 0.676 |
| No | 879 | 745(90.0) | | 134(90.5) |  |  |  |
| Age |  |  | |  |  |  |  |
| ≤19 years | 73 | 61(7.4) | | 12(8.1) | 1.06 | 0.55-2.06 | 0.854 |
| >19 years | 902 | 766(92.6) | | 136(91.9) |  |  |  |
| Parity |  |  | |  |  |  |  |
| Primiparous | 463 | 386(46.6) | | 77(52.0) | 1.39 | 0.92-2.10 | 0.115 |
| Multiparous | 513 | 442(53.4) | | 71(48.9) |  |  |  |
| Past history of gestational hypertension |  |  | |  |  |  |  |
| Yes | 34 | 26(3.1) | | 8(5.4) | 1.77 | 0.74-4.19 | 0.196 |
| No | 942 | 802(96.9) | | 140(94.6) |  |  |  |
| Past history of chronic hypertension |  |  | |  |  |  |  |
| Yes | 8 | 6(0.7) | | 2(1.4) | 1.45 | 0.26-8.10 | 0.670 |
| No | 965 | 820(99.3) | | 145(98.6) |  |  |  |
| ECLAMPSIA/PRE-ECLAMPSIA | | | | | | | |
| Yes | 25 | 17(2.1) | | 8(5.4) | 0.41 | .17-0.99 | **0.049** |
| No | 951 | 811(97.9) | | 140(94.6) |  |  |  |
| Age |  |  | |  |  |  |  |
| ≤19 years | 73 | 61(7.4) | | 12(8.1) | 1.00 | 0.52-1.96 | 0.984 |
| >19 years | 902 | 766(92.6) | | 136(91.9) |  |  |  |
| Parity |  |  | |  |  |  |  |
| Primiparous | 463 | 386(46.6) | | 77(52.0) | 1.35 | 0.90-2.04 | 0.149 |
| Multiparous | 513 | 442(53.4) | | 71(48.9) |  |  |  |
| Past history of gestational hypertension |  |  | |  |  |  |  |
| Yes | 34 | 26(3.1) | | 8(5.4) | 1.63 | 0.68-3.90 | 0.272 |
| No | 942 | 802(96.9) | | 140(94.6) |  |  |  |
| Past history of chronic hypertension |  |  | |  |  |  |  |
| Yes | 8 | 6(0.7) | | 2(1.4) | 1.19 | 0.21-6.77 | 0.843 |
| No | 965 | 820(99.3) | | 145(98.6) |  |  |  |
| PERINEAL TEARS | | | | | | | |
| Yes | 65 | 59(7.1) | | 6(4.1) | 1.895 | 0.80-4.50 | 0.147 |
| No | 911 | 769(92.9) | | 142(95.9) |  |  |  |
| Age |  |  | |  |  |  |  |
| ≤19 years | 73 | 61(7.4) | | 12(8.1) | 1.07 | 0.55-2.06 | 0.849 |
| >19 years | 902 | 766(92.6) | | 136(91.9) |  |  |  |
| Parity |  |  | |  |  |  |  |
| Primiparous | 463 | 386(46.6) | | 77(52.0) | 1.42 | 0.94-2.14 | 0.092 |
| Multiparous | 513 | 442(53.4) | | 71(48.9) |  |  |  |
| Past history of gestational hypertension |  |  | |  |  |  |  |
| Yes | 34 | 26(3.1) | | 8(5.4) | 1.71 | 0.72-4.08 | 0.224 |
| No | 942 | 802(96.9) | | 140(94.6) |  |  |  |
| Past history of chronic hypertension |  |  | |  |  |  |  |
| Yes | 8 | 6(0.7) | | 2(1.4) | 1.50 | 0.27-8.48 | 0.643 |
| No | 965 | 820(99.3) | | 145(98.6) |  |  |  |

**Supplementary table 1b: Details of multivariate analysis (b)**

| Dependent variables and covariates | checklist use (SCC) | | | Adjusted OR | CI (95%) | P-value |
| --- | --- | --- | --- | --- | --- | --- |
|  | **Total** | **Yes** | **No** |  |  |  |
| NEONATAL COMPLICATIONS | | | | | | |
| Yes | 85 | 70(8.5) | 15(10.2) | 0.81 | 0.45-1.46 | 0.487 |
| No | 889 | 757(91.5) | 132(89.8) |  |  |  |
| Age |  |  |  |  |  |  |
| ≤19 years | 73 | 61(7.4) | 12(8.1) | 1.05 | 0.54-2.02 | 0.885 |
| >19 years | 902 | 766(92.6) | 136(91.9) |  |  |  |
| Parity |  |  |  |  |  |  |
| Primiparous | 463 | 386(46.6) | 77(52.0) | 1.40 | 0.93-2.12 | 0.106 |
| Multiparous | 513 | 442(53.4) | 71(48.9) |  |  |  |
| Past history of gestational hypertension |  |  |  |  |  |  |
| Yes | 34 | 26(3.1) | 8(5.4) | 1.80 | 0.76-4.26 | 0.184 |
| No | 942 | 802(96.9) | 140(94.6) |  |  |  |
| Past history of chronic hypertension |  |  |  |  |  |  |
| Yes | 8 | 6(0.7) | 2(1.4) | 1.45 | 0.26-8.07 | 0.670 |
| No | 965 | 820(99.3) | 145(98.6) |  |  |  |
| NEONATAL ASPHYXIA | | | | | | |
| Yes | 45 | 37(4.5) | 8(5.4) | 0.81 | 0.45-1.46 | 0.487 |
| No | 930 | 790(95.5) | 140(94.6) |  |  |  |
| Age |  |  |  |  |  |  |
| ≤19 years | 73 | 61(7.4) | 12(8.1) | 1.05 | 0.54-2.03 | 0.879 |
| >19 years | 902 | 766(92.6) | 136(91.9) |  |  |  |
| Parity |  |  |  |  |  |  |
| Primiparous | 463 | 386(46.6) | 77(52.0) | 1.37 | 0.91-2.06 | 0.132 |
| Multiparous | 513 | 442(53.4) | 71(48.9) |  |  |  |
| Past history of gestational hypertension |  |  |  |  |  |  |
| Yes | 34 | 26(3.1) | 8(5.4) | 1.77 | 0.75-4.21 | 0.193 |
| No | 942 | 802(96.9) | 140(94.6) |  |  |  |
| Past history of chronic hypertension |  |  |  |  |  |  |
| Yes | 8 | 6(0.7) | 2(1.4) | 1.43 | 0.26-7.95 | 0.681 |
| No | 965 | 820(99.3) | 145(98.6) |  |  |  |
| STILL BIRTH | | | | | | |
| Yes | 40 | 33(4.0) | 7(4.7) | 0.809 | 0.37-1.78 | 0.598 |
| No | 935 | 794(96.0) | 141(95.3) |  |  |  |
| Age |  |  |  |  |  |  |
| ≤19 years | 73 | 61(7.4) | 12(8.1) | 1.05 | 0.55-2.04 | 0.7872 |
| >19 years | 902 | 766(92.6) | 136(91.9) |  |  |  |
| Parity |  |  |  |  |  |  |
| Primiparous | 463 | 386(46.6) | 77(52.0) | 1.37 | 0.91-2.06 | 0.132 |
| Multiparous | 513 | 442(53.4) | 71(48.9) |  |  |  |
| Past history of gestational hypertension |  |  |  |  |  |  |
| Yes | 34 | 26(3.1) | 8(5.4) | 1.76 | 0.74-4.17 | 0.199 |
| No | 942 | 802(96.9) | 140(94.6) |  |  |  |
| Past history of chronic hypertension |  |  |  |  |  |  |
| Yes | 8 | 6(0.7) | 2(1.4) | 1.44 | 0.26-7.97 | 0.679 |
| No | 965 | 820(99.3) | 145(98.6) |  |  |  |
| NEONATAL DEATHS | | | | | | |
| Yes | 3 | 2(0.2) | 1(0.7) | 0.375 | 0.03-4.18 | 0.425 |
| No | 972 | 825(99.8) | 147(99.3) |  |  |  |
| Age |  |  |  |  |  |  |
| ≤19 years | 73 | 61(7.4) | 12(8.1) | 1.06 | 0.55-2.06 | 0.853 |
| >19 years | 902 | 766(92.6) | 136(91.9) |  |  |  |
| Parity |  |  |  |  |  |  |
| Primiparous | 463 | 386(46.6) | 77(52.0) | 1.36 | 0.91-2.05 | 0.137 |
| Multiparous | 513 | 442(53.4) | 71(48.9) |  |  |  |
| Past history of gestational hypertension |  |  |  |  |  |  |
| Yes | 34 | 26(3.1) | 8(5.4) | 1.76 | 0.74-4.18 | 0.198 |
| No | 942 | 802(96.9) | 140(94.6) |  |  |  |
| Past history of chronic hypertension |  |  |  |  |  |  |
| Yes | 8 | 6(0.7) | 2(1.4) | 1.43 | 0.26-7.93 | 0.686 |
| No | 965 | 820(99.3) | 145(98.6) |  |  |  |
